# Chronic Kidney Disease and Cerebrovascular Pathology: Incidence and Functional Outcomes in Riga East University Hospital

**DOI:** 10.3390/medicina60020219

**Published:** 2024-01-27

**Authors:** Violeta Zubkova, Aleksejs Ševčenko, Igors Miļuhins, Ilga Ķikule, Iveta Haritončenko, Guntis Karelis

**Affiliations:** 1 Stroke Unit, Neurovascular Department, Riga East University Hospital, 1038 Riga, Latvia; ilga.kikule@gmail.com (I.Ķ.); iveta.hari@gmail.com (I.H.); guntis.karelis@gmail.com (G.K.); 2 Neurology and Neurosurgery Department, Rīga Stradiņš University, 1007 Riga, Latvia; aleksejs.sevcenko@inbox.lv (A.Š.); igormiluhin@gmail.com (I.M.); 3 Infectology Department, Rīga Stradiņš University, 1007 Riga, Latvia

**Keywords:** stroke, ischemic stroke, hemorrhagic stroke, chronic kidney disease, functional outcome, secondary prevention

## Abstract

*Background and Objectives*: The aim of this study was to investigate the incidence of cerebrovascular pathology in patients with chronic kidney disease and its effect on functional outcomes. *Materials and Methods*: In a retrospective cross-sectional study (2018–2021), the medical records of patients with acute hemorrhagic and ischemic stroke with concomitant chronic kidney disease who received treatment in Riga East University Hospital Stroke Unit were analyzed. Data were analyzed using IBM SPSS 26.0. The Kruskal–Wallis, Mann–Whitney U test, and Spearman’s rank correlation coefficient methods were used. *Results*: The final sample consisted of 305 acute cerebrovascular pathology patients (56.4% females). Overall, 57.3% of stroke patients had second-stage chronic kidney disease with average serum creatinine levels of 104.3 mmol/L (±32.8). The functional outcome of the stroke depended on the stage of chronic kidney disease. There was a statistically significant non-linear correlation between glomerular filtration rate and NIHSS (National Institute of Health Stroke Scale) score on admission (Rho −0.194, *p* = 0.016), glomerular filtration rate and NIHSS score on discharge (Rho −0.186, *p* = 0.020), and glomerular filtration rate and modified Rankin score on admission (Rho −0.237, *p* = 0.003) and discharge (Rho −0.224, *p* = 0.05). The mean NIHSS score of ischemic stroke patients was 8.3 ± 5.9 on admission and 6.5 ± 5.8 on discharge. In the hemorrhagic stroke patient group, the mean NIHSS score was 9.5 ± 7.3 on admission and 7.1 ± 6.9 on discharge. On average, 34.0% of ischemic stroke patients had an mRS score of 5 on admission, while in the hemorrhagic stroke patient group, this figure was 41%. There was no statistical difference in the glomerular filtration rate between the thrombolyzed versus non-thrombolyzed patient groups (Mann–Whitney *U* test = 1457, *p* = 0.794). *Conclusions*: Chronic kidney disease is an important predictor of the severity and functional outcome of a stroke; furthermore, the early management and prevention of complications should be a top priority in the prophylaxis of this cerebrovascular pathology.

## 1. Introduction

The incidence of chronic kidney disease worldwide is approximately 13.4%, making it one of the most common chronic conditions in adults and the elderly [1]. Chronic kidney disease is known to be an independent risk factor for ischemic and hemorrhagic stroke; it is associated with a greater neurological deficit following ischemic stroke, a poor functional outcome, and greater mortality [2]. The co-existence of vascular risk factors, such as hypertension, diabetes mellitus, dyslipidemia, proteinuria, accelerated atherosclerosis, vascular calcification, the effect of uremic toxins, prothrombotic tendency, and impaired cerebral autoregulation, is highly prevalent in chronic kidney disease patients [3]. The estimated risk of stroke in patients with end-stage kidney disease is 5–30 times greater than in the general population [4]. Chronic kidney disease (CKD) is known to have an impact on treatment strategy as well as secondary prophylaxis. There is an inequality in the management of acute stroke in patients with chronic kidney disease—both in the United States and Europe [5,6]. In an analysis of nearly 700,000 patients from the Get With The Guideline—Stroke program cohort, patients with chronic kidney disease were less likely to receive evidence-based therapies compared with no chronic kidney disease, such as thrombolytic therapy, antiplatelets, statins, and smoking cessation programs [5]. Furthermore, stroke can influence daily activities and quality of life after the acute cerebrovascular event. In the Choices for Healthy Outcomes in Caring for End-Stage Renal Disease (CHOICE) study of patients receiving dialysis, only 56% of patients were able to be discharged to their home or acute rehabilitation [7]. There are few studies available on the functional neurological outcomes of acute stroke in patients with concomitant chronic kidney disease. The aim of this study was to investigate the incidence, functional outcome, stroke severity, and secondary prevention of ischemic and hemorrhagic stroke in patients with concomitant chronic kidney disease.

## 2. Materials and Methods

In this retrospective cross-sectional study (2018–2021), medical records of acute stroke patients with concomitant chronic kidney disease were analyzed.

### 2.1. Study Population

This study utilized the data collected from the patients admitted to the Neurovascular Department of the Riga East University Hospital, Riga, Latvia from January 2018 to January 2021 who were diagnosed with acute ischemic stroke or hemorrhagic stroke and concomitant chronic kidney disease. In this study, patients were older than 18 years.

### 2.2. Measures and Definitions

All the patients included in this study were divided into 2 main groups according to the pathogenesis of the acute stroke (stroke onset less than 7 days)—either ischemic or hemorrhagic stroke. If the patients had an ischemic stroke, they were further subdivided according to the Trial of Org 10172 in Acute Stroke Treatment (TOAST) classification [8]. If the patients had a hemorrhagic stroke, then they were divided by the localization of the hemorrhage into either intracerebral hematoma or subarachnoid hemorrhage and further subdivided according to the cause of the hemorrhage: hypertensive, aneurysm rupture, anticoagulation-related, vasculitis, arterio-venous malformation, or cryptogenic.

Chronic kidney disease was defined according to the KDIGO (Kidney Disease: Improving Global Outcomes) definition as an abnormality of the kidney structure or function for at least three months or a glomerular filtration rate less than 60 mL/min for at least three months with or without evidence of kidney damage, which could present as proteinuria, hematuria, or pathological changes in histology or imaging. The severity of chronic kidney disease was classified into five stages based on glomerular filtration rate [9]. Patients with a normal or mildly decreased glomerular filtration rate (GFR 60–90 mL/min) were considered for inclusion. This encompassed individuals exhibiting signs of nephrotic or nephritic syndrome, those with asymptomatic urine analysis results, and those with asymptomatic pathological kidney imaging findings. Additionally, patients experiencing hypertension attributable to kidney disease were included. According to KDIGO guidelines, such patients were classified as having stage 1 or stage 2 chronic kidney disease [9].

Patients with a glomerular filtration rate of 90 mL/min or in the range 60–90 mL/min and no other signs of chronic kidney damage, e.g., proteinuria, hematuria, pathological kidney biopsy or imaging results, as well as patients with a first-time documented decrease in glomerular filtration rate and patients with acute kidney failure, were excluded from this study.

### 2.3. Clinical Course and Functional Outcome

In this study, acute stroke patients with a stroke onset of less than 7 days were included. The functional outcome of the cerebrovascular pathology was estimated using the National Institutes of Health Stroke Scale (NIHSS) and the modified Rankin scale (mRS) on admission to the hospital and discharge.

The National Institutes of Health Stroke Scale (NIHSS) is a comprehensive 15-item scale designed to assess the severity of a stroke. It evaluates various domains, including the level of consciousness, eye movements, visual field integrity, facial movements, muscle strength in the arms and legs, sensation, coordination, language, speech, and neglect [10].

The modified Rankin score (mRS) assessment gauges post-stroke functional independence concerning pre-stroke activities through a single-scale item. Disability is categorized as follows: none, slight, moderate, moderately severe, and severe. A score of 0 refers to no symptoms at all [11].

### 2.4. Statistical Analysis

Data were analyzed using IBM SPSS 26.0. Non-parametric tests—Kruskal–Wallis, Mann–Whitney U test, and Spearman’s rank correlation coefficient—were used. A *p*-value of <0.05 was considered statistically significant.

## 3. Results

### 3.1. General Characteristics

The final sample consisted of 305 acute cerebrovascular pathology patients; 155 were acute ischemic stroke patients, while the other half were hemorrhagic. In the ischemic stroke patient group, 62.6% were females, 37.4% were males, and the mean age was 76 ± 11.2 years (Table 1). 

### 3.2. Ischemic Stroke and Chronic Kidney Disease

**Figure 1:**
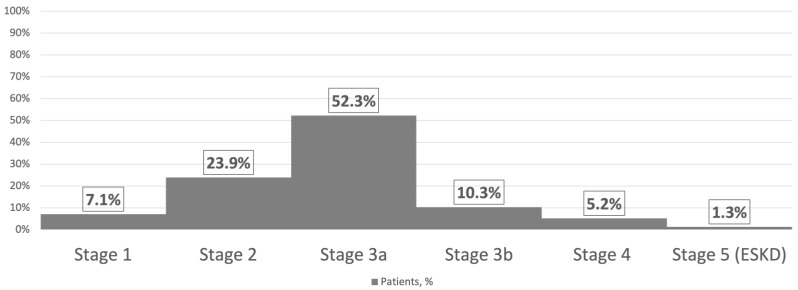
Stages of chronic kidney disease in ischemic stroke patients, n = 155.

In total, 51.6% of patients experienced a cardioembolic stroke, 21.9% experienced an atherothrombotic stroke, and 26.5% experienced a cryptogenic stroke by etiology (TOAST classification) [2]. For ischemic stroke, 80.0% of cases were localized in the anterior circulation; of these, 45.8% were in the left middle cerebral artery, while 20.0% of patients had a stroke in the posterior circulation. Most patients (52.3%) had stage 3a chronic kidney disease, 23.9% had stage 2, 10.3% had stage 3b, 7.1% had stage 1, 5.2% had stage 4, and 1.3% had stage 5 (end-stage kidney disease) (Figure 1). 

The mean serum creatinine levels were 99.4 ± 43.7 µmol/L, and the mean glomerular filtration rate (GFR) was 59.86 ± 20.1 mL/min. Patients with a GFR value exceeding 30 are eligible to receive intravenous iodinated contrast. The modified Rankin score on admission in 33.5% of cases was five, and in 26.5%, it was four on discharge (Table 2). 

Furthermore, there was a statistically significant correlation between glomerular filtration rate and mRS on admission (Rho −0.237, *p* = 0.003) as well as on discharge (Rho −0.224, *p* = 0.05). The NIHSS scores were 8 ± 5.9 on admission and 6.5 ± 5.8 on discharge, and there was a statistically significant non-linear correlation between GFR and the rates of this score both on admission (Rho −0.194, *p* = 0.016) and on discharge (Rho −0.186, *p* = 0.020). This study included 86.5% of patients who received thrombolytic therapy as a remedial drug in acute stroke settings; nevertheless, there was no statistical difference between patients who received thrombolysis or not based on their GFR (Mann–Whitney *U* test = 1457, *p* = 0.794).

The majority of patients with chronic kidney disease received secondary prophylaxis after an acute ischemic event, and GFR was statistically significantly associated with the type of the received anticoagulant; the lower GFR was, the more frequently patients were prescribed edoxaban as a secondary prophylaxis for cardioembolic stroke (Kruskal–Wallis, *p* = 0.007), except for end-stage chronic kidney disease patients (*note*: *the administration of edoxaban is contraindicated if GFR is less than 15 mL/min*). The lower the glomerular filtration rate a patient had, the more often they received an adjusted dose of oral anticoagulant. A total of 43.2% of patients with an atherothrombotic or cryptogenic cause of a stroke received acetylsalicylic acid as secondary stroke prevention. In comparison, clopidogrel was prescribed in 3.9% of patients, and 5.8% of these patients received dual anti-aggregation therapy.

The majority (49.0%) of chronic kidney disease patients had second-stage primary arterial hypertension (Table 1) and were discharged on angiotensin-converting enzyme inhibitors (65.2%), calcium channel blockers (49.7%), and the diuretic, antihypertensive drug class (47.7%). Less frequently, patients were prescribed angiotensin II receptor blockers (14.9%) and alpha 2 receptor blockers (19.4%) (Table 3).

Overall, 85.1% of the ischemic stroke patients were prescribed statin therapy, regardless of the stroke etiology. Out of 155 ischemic stroke patients, 71 received a 20-mg dosage of a lipid-lowering drug (either atorvastatin or rosuvastatin) as the secondary stroke prophylaxis.

### 3.3. Hemorrhagic Stroke and Chronic Kidney Disease

**Figure 2:**
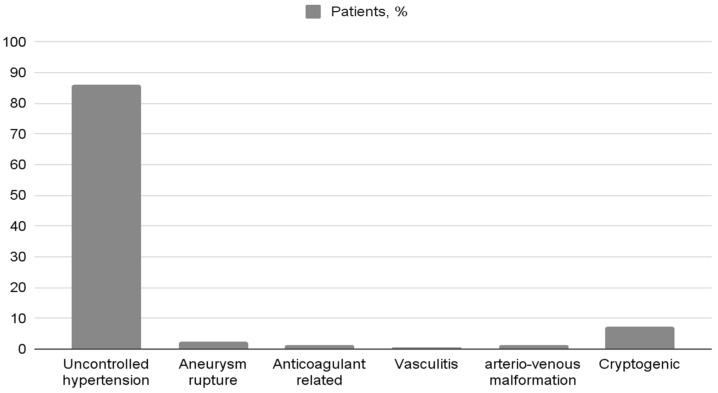
The etiology of hemorrhagic stroke, n = 150.

In the hemorrhagic stroke patient group, 51.0% were females, 49.0% were males, and the mean age was 71.7 ± 14.4 years (Table 1). In total, 82.6% of these patients had spontaneous intracerebral hemorrhage; in 86.0% of cases, this was caused by uncontrolled hypertension; in 2.6% by the rupture of an aneurysm; in 1.3%, this was related to anticoagulation therapy; in 1.3% due to vasculitis; in 1.3% due to arterio-venous malformation; and in 7.3% of cases due to cryptogenic etiology (Figure 2).

**Figure 3:**
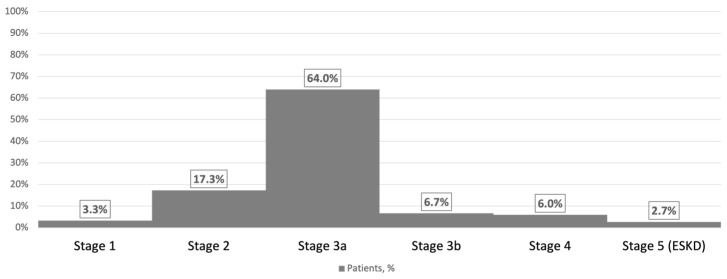
The stages of chronic kidney disease in hemorrhagic stroke patients, n = 150.

In most cases (59.3%), the hemorrhage was localized in the right hemisphere. Of these cases, 64.0% had stage 3a chronic kidney disease, 17.3% had stage 2, 6.7% had stage 3b, 6.0% had stage 4, 3.3% had stage 1, and 2.7% had end-stage kidney disease (Figure 3).

The mean glomerular filtration rate was 55.0 ± 21.9 mL/min, and serum creatinine levels were 109.1 ± 85.4 mmol/L. The modified Rankin score on admission was 4 (40.7%), while on discharge it was 3 (26.7%) (Table 4).

The average NIHSS score on admission was 9.5 ± 7.3, while on discharge it was 7.1 ± 6.9. In hemorrhagic stroke, a statistically significant correlation was not found between glomerular filtration rate and mRS on admission (Rho 0.104, *p* = 0.209) and discharge (Rho 0.040, *p* = 0.625), or between glomerular filtration rate and NIHSS score on admission (Rho −0.041, *p* = 0.623) and discharge (Rho 0.003, *p* = 0.976).

In total, 57.0% of hemorrhagic stroke patients had third-degree primary arterial hypertension (Table 1) and were discharged on angiotensin-converting enzyme inhibitors (71.3%), calcium channel blockers (60.7%), the diuretic, antihypertensive drug class (41.3%), angiotensin II receptor blockers (14.0%), or alpha 2 receptor blockers (19.4%) (Table 5).

In the hemorrhagic stroke patient group, a statistically significant correlation was found between the glomerular filtration rate and the type of prescribed antihypertensive drug. The glomerular filtration rate was 10 mL/min lower in those patients who received antihypertensives of the beta-blocker drug class. In comparison, GFR was 10 mL/min higher in those who received angiotensin receptor blockers.

Regarding statin therapy, only 14.0% of hemorrhagic stroke patients received it as secondary stroke prophylaxis.

## 4. Discussion

The aim of this study was to investigate the incidence of cerebrovascular pathology in patients with chronic kidney disease and its effect on functional outcomes. The study involved 305 patients with acute cerebral pathology, where half were patients with acute ischemic stroke and the remainder consisted of patients with hemorrhagic stroke. Patients were mainly females with a mean age of approximately 70 years. As mentioned, the highest number of patients had stage 3a kidney disease, which is characterized by GFR rates of 45–59 mL/min. In the ischemic stroke patient group, 51.6% had a cardioembolic etiology according to the TOAST classification [8]. Similarly, in a study published by Seon-Mi K. et al. (2023), higher stages of chronic kidney disease were associated with a higher risk of atrial fibrillation development. The incidence rates of atrial fibrillation were 1.77 per 1000 person-years for subjects without chronic kidney disease and 4.33 for those with stage 4 CKD [12].

According to the National Kidney Foundation, CKD is defined as GFR < 60 mL/min/1.73 m^2^ or persistent kidney damage markers in a term longer than three months [13]. Observing the same GFR level in each group, the creatinine level was higher in the hemorrhagic stroke patient group. MRS was higher in the ischemic stroke group compared to the hemorrhagic stroke group on admission and discharge, but the opposite was seen for the NIHSS score. Higher mRS indicates more severe functional impairment, which applies to the NIHSS. To sum up, interpreting a stroke’s severity may depend on the scale used in a case. Chronic kidney disease patients have 49.0% greater risk of neurological deterioration, defined as a two-point increase in the NIHSS scale, and 25.0% greater risk of a mRS of 2 or more at discharge than patients without chronic kidney disease [3,14]. In a study conducted by Nagaraja et al. (2023), in patients with kidney function impairment, higher NIHSS scores were observed, showing a higher neurological deficit compared to the control group without a loss of kidney function [15]. A correlation was observed between GFR and mRS on admission and discharge and NIHSS score on admission and discharge in ischemic patient groups. In a study conducted by Fabjan et al. (2007), it was observed that in patients with acute ischemic stroke, patient mortality was higher in patients with a higher NIHSS score, lower GFR, and lower albumin levels [16]. Our study observed no exact correlation between GFR, NIHSS score, and mRS in a hemorrhagic stroke patient group. In a study conducted by Zhaoxia et al. (2022), it was found that the risk of in-hospital mortality, non-routine discharge, hemorrhagic stroke severity, and in-hospital stroke complications increased as the glomerular filtration rate declined [17]. Another study conducted by Yang et al. (2014) revealed no correlation between glomerular filtration rate and increased risk of disability at 3 months [18]. The data discrepancy may be related to the differences in the study design and the study population.

Irrespective of age and stroke severity, intravenous thrombolysis administered within a time window is associated with better functional outcomes [19], which is also crucial for chronic kidney disease patients, who are often prone to large cerebral infarcts due to the high prevalence of atrial fibrillation [20]. Our study included 86.5% of patients who received thrombolytic therapy as a remedial drug in acute stroke settings; nevertheless, there was no statistical difference between patients who received thrombolysis or not based on their GFR. The use of intravenous thrombolysis in CKD patients is one of the key inequities in stroke care, with delays to treatment [21] and under-treatment reported due to the assumed higher risk of intracerebral hemorrhage [5,6]. Although there still have not been enough studies conducted in this field, the guidelines consider it safe to use intravenous thrombolysis in otherwise-eligible patients with chronic kidney disease, including hemodialysis patients with a normal activated partial thromboplastin time [22].

According to the data, patients in the ischemic stroke group mostly had second- and third-stage primary arterial hypertension. In the hemorrhagic stroke group, more than eighty percent of cases of stroke were caused by uncontrolled arterial hypertension. Hence, control of hypertension is one of the primary goals of patients who have survived a cerebrovascular event. According to data provided by Kelly et al., hypertension is prevalent in a substantial percentage, ranging from 67.0% to 92.0%, of individuals with CKD. Recent findings indicate that adjusting for long-term premorbid blood pressure significantly complicates the relationship between CKD and stroke. Current evidence suggests that CKD is linked to an increased tendency toward thrombosis. However, the clots formed in CKD exhibit distinct structural and functional characteristics compared to those in individuals with normal renal function. This divergence may elucidate why individuals with CKD face a heightened risk of thrombosis while paradoxically being at an elevated risk of bleeding [23].

The most frequently prescribed antihypertensive medicine was ACEIs. In the context of preventing secondary strokes, the effectiveness of diuretics and an ACEI is notable, and they complement other therapies aimed at reducing atherogenic and thrombogenic risks, including the use of aspirin [24]. In a hemorrhagic stroke group, a correlation was found between the use of antihypertensives and GFR. In patients who used ARBs, GFR was 10 mL/min higher. Both medications affect the renin–angiotensin–aldosterone system differently, where ARBs block angiotensin II type 1 receptors and ACEIs inhibit an angiotensin-converting enzyme. A study provided by Ma et al. (2012) shows that patients who use ACEIs have a lower risk of non-fatal stroke compared to the patient group that uses ARBs [25]. Atherosclerosis is a basic pathological factor for atherothrombotic brain infarction, whereas arteriosclerosis is a factor for intraparenchymal hemorrhage [26]. Endothelial dysfunction is common in CKD in which uremic toxins, insulin resistance, vascular calcification, dyslipidemia, anemia, and renin–angiotensin activation are proposed to cause chronic inflammation and oxidative stress and promote atherogenesis and arteriosclerosis. Stroke risk increases when a decline in GFR and an increase in albuminuria are observed. A GFR of <90 mL/min/1.73 m^2^ is associated with an increased risk of all-cause stroke by 39.0%. The Kidney Disease: Improving Global Outcomes (KDIGO) classification of CKD may be a useful tool for stratifying the risk of stroke in the general population. However, estimates of the absolute risk of stroke require adjustment for race and clinical setting [27]. Hypertension can be defined as an independent risk factor for stroke and chronic kidney disease. Long-term blood pressure control can be defined as a potentially important confounder of the association between chronic kidney disease and stroke risk [28].

Statin therapy is beneficial in reducing the risk of cardiovascular events in non-dialysis chronic kidney disease patients [29]. Our results revealed that statin therapy was prescribed for 85.1% of ischemic stroke patients, while it was prescribed for only 14.0% of patients in the hemorrhagic stroke patient group. Similar results were found in the Salfort kidney cohort study (2019)—statin therapy was prescribed for 75.0% of ischemic stroke patients [30].

The limitations of this study include a retrospective design with no opportunity to collect data directly from patients included in the study. Additionally, there was no opportunity for long-term follow-up with patients after discharge to determine functional outcomes. Data records that were provided sometimes lacked crucial information, so there was a reason to exclude some potential participants from the study. No additional factors that could have influenced functional outcomes were explored. 

Another limitation arises from the insufficient information available on specific causes of CKD, such as diabetic nephropathy, hypertensive nephrosclerosis, and glomerulonephritis (GN), with a particular emphasis on drug-induced GN. In a study conducted by Kaze et al., in a group of 9170 participants with type 2 diabetes that was followed for 4.9 years, 156 participants developed a stroke. Compared with participants with both normal urine Albumin–Creatinine Ratio (UACR) and eGFR, those with UACR ≥ 30 mg/g and eGFR < 60 mL/min/1.73 m^2^ had increased risks of stroke [31].

## 5. Conclusions

A large sample of patients (n = 305) was investigated retrospectively for three years, observing the correlations of different stroke etiologies with functional outcomes in patients with impaired renal function. Chronic kidney disease was an essential predictor of the severity of cerebrovascular pathology; more specifically, it had a high impact on the functional outcome in the ischemic stroke patient group. A statistically significant correlation was found between glomerular filtration rate on admission and discharge and NIHSS and modified Rankin scores, and consequently, lower glomerular filtration was the worse functional outcome in ischemic stroke patients. Compared to the hemorrhagic stroke patient group, no statistically significant correlation was found between glomerular filtration rate and functional outcome. More prospective multicenter studies should be conducted to evaluate the CKD effect on functional outcomes in patients after stroke. Evaluating a functional outcome over a longer period can be considered for future research. It is important to prevent CKD progression and improve post-stroke functional outcomes by controlling risk factors such as hypertension, which can affect CKD progression and hemorrhagic stroke incidence, and diabetes, which is one of the main risk factors for CKD and hyperlipidemia.

## Figures and Tables

**Table 1:** Demography of ischemic stroke patient and hemorrhagic stroke patient group, n = 305.

		Ischemic Stroke Patients n = 155	Hemorrhagic Stroke Patients n = 150
Gender	Females (%)	62.6	51.0
	Males (%)	37.4	49.0
Mean age (years)		76 ± 11.2	71.7 ± 14
Blood pressure (BP) category	Normal (%)	6.2	4.5
	Elevated (%)	7.2	4.5
	Stage 1 hypertension (%)	5.6	11
	Stage 2 hypertension (%)	49	23
	Stage 3 hypertension (%)	32	57

**Table 2:** The modified Rankin score (mRS) on admission and discharge in ischemic stroke patients n = 155.

Modified Rankin Score (mRS)	On Admission, %n = 155	On Discharge, %n = 155
0	1.9	5.2
1	7.1	11.6
2	4.5	14.2
3	18.1	19.4
4	34.8	26.5
5	33.5	6.0
6	0.0	1.0

**Table 3:** Prescribed antihypertensive drugs in ischemic stroke patient group, n = 155.

Antihypertensive Drug Class	Patients, %n = 155
Diuretics	47.7
Alpha 2 receptor blockers	15.5
Beta-blockers	19.4
Angiotensin II receptor blockers	14.8
Angiotensin-converting enzyme (ACE) inhibitors	65.2
Ca^2+^ channel blockers	49.8

**Table 4:** The modified Rankin scores (mRS) on admission and discharge in hemorrhagic stroke patients n = 150.

Modified Rankin Score (mRS)	On Admission, %n = 150	On Discharge, %n = 150
0	0.0	0.0
1	4.7	8.0
2	8.7	17.3
3	14.7	18.7
4	28.8	26.7
5	40.7	19.3
6	1.3	6.0

**Table 5:** Prescribed antihypertensive drugs in hemorrhagic stroke patient group n = 150.

Antihypertensive Drug Class	Patients, %n = 150
Diuretics	41.3
Alpha 2 receptor blockers	44.0
Beta-blockers	54.6
Angiotensin II receptor blockers	14.0
Angiotensin-converting enzyme (ACE) inhibitors	71.3
Ca^2+^ channel blockers	60.7

## Data Availability

Data are available upon request due to ethical restrictions. All the data included in this study are available upon request from the corresponding author. The data are not publicly available and are stored in the patient medical record repository at Riga East University Hospital.

## References

[1] Kovesdy C.P. (2022). Epidemiology of Chronic Kidney Disease: An update 2022. Kidney Int. Suppl..

[2] Ovbiagele B., Sanossian N., Liebeskind D.S., Kim D., Ali L.K., Pineda S., Saver J.L. (2009). Indices of Kidney Dysfunction and Discharge Outcomes in Hospitalized Stroke Patients without Known Renal Disease. Cerebrovasc. Dis..

[3] Nayak-Rao S., Shenoy M.P. (2017). Stroke in Patients with Chronic Kidney Disease…: How do we Approach and Manage it?. Indian J. Nephrol..

[4] Longenecker J.C., Coresh J., Powe N.R., Levey A.S., Fink N.E., Martin A., Klag M.J. (2002). Traditional Cardiovascular Disease Risk Factors in Dialysis Patients Compared with the General Population: The CHOICE Study. J. Am. Soc. Nephrol..

[5] Ovbiagele B., Schwamm L.H., Smith E.E., Grau-Sepulveda M.V., Saver J.L., Bhatt D.L., Hernandez A.F., Peterson E.D., Fonarow G.C. (2014). Patterns of Care Quality and Prognosis among Hospitalized Ischemic Stroke Patients with Chronic Kidney Disease. J. Am. Heart Assoc..

[6] Findlay M.D., Dawson J., MacIsaac R., Jardine A.G., MacLeod M.J., Metcalfe W., Traynor J.P., Mark P.B. (2018). Inequality in care and Differences in Outcome Following Stroke in People with ESRD. Kidney Int. Rep..

[7] Sozio S.M., Armstrong P.A., Coresh J., Jaar B.G., Fink N.E., Plantinga L.C., Powe N.R., Parekh R.S. (2009). Cerebrovascular Disease Incidence, Characteristics, and Outcomes in Patients Initiating Dialysis: The Choices for Healthy Outcomes in Caring for ESRD (CHOICE) study. Am. J. Kidney Dis..

[8] Adams H.P., Bendixen B.H., Kappelle L.J., Biller J., Love B.B., Gordon D.L. (1993). Marshard E.E. Classification of Subtype of Acute Ischemic Stroke. Definitions for Use in a Multicenter Clinical Trial. TOAST. Trial of Org 10172 in Acute Stroke Treatment. Stroke.

[9] Levin A., Stevens P., Bilous R., Coresh J. (2013). Kidney Disease: Improving Global Outcomes 2012 Clinical Practice Guidelines for the Evaluation and Management of Chronic Kidney Disease. Off. J. Int. Soc. Nephrol..

[10] Kwah L.K., Diong J. (2014). National Institutes of Health Stroke Scale (NIHSS). J. Physiother..

[11] Wilson J.T., Hareendran A., Grant M., Baird T., Schulz U.G., Muir K.W., Bone I. (2002). Improving the Assessment of Outcomes in Stroke: Use of a Structured Interview to Assign Grades on the Modified Rankin Scale. Stroke.

[12] Kim S.-M., Jeong Y., Kim Y.L., Kang M., Kang E., Ryu H., Kim Y., Han S.S., Ahn C., Oh K.-H. (2023). Association of Chronic Kidney Disease with Atrial Fibrillation in General Adult Population: A Nationwide Population Based Study. J. Am. Heart Assoc..

[13] Vassalotti J.A., Centor R., Turner B.J., Greer R.C., Choi M., Sequist T.D. (2016). Practical Approach to Detection and Management of Chronic Kidney Disease for the Primary Care Clinician. Am. J. Med..

[14] Kumai Y., Kamouchi M., Hata J., Ago T., Kitayama J., Nakane H., Sugimori H., Kitazono T., FSR Investigators (2012). Proteinuria and Clinical Outcomes after Ischemic Stroke. Neurology.

[15] Nagaraja N., Farooqui A., Ballur Narayana Reddy V., Shukla A.M. (2023). Kidney Impairment and Outcomes in Acute Ischaemic Stroke. Intern. Med. J..

[16] Hojs Fabjan T., Hojs R., Tetickovic E., Pecovnik Balon B. (2007). Ischaemic Stroke—The Impact of Renal Dysfunction on in-hospital Mortality. Eur. J. Neurol..

[17] Li Z., Li Z., Zhou Q., Gu H., Wang Y., Zhao X. (2022). Effects of Estimated Glomerular Filtration Rate on Clinical Outcomes in Patients with Intracerebral hemorrhage. BMC Neurol..

[18] Yang J., Arima H., Zhou J., Zhao Y., Li Q., Wu G., Zhang Y. (2014). Effects of Low Estimated Glomerular Filtration Rate on Outcomes after Stroke: A Hospital—Based Stroke Registry in China. Eur. J. Neurol..

[19] Emberson J., Lees K.R., Lyden P., Blackwell L., Albers G., Bluhmki E., Brott T., Cohen G., Davis S., Donnan G. (2014). Effect of treatment delay, age, and stroke severity on the effects of intravenous thrombolysis with alteplase for acute ischaemic stroke: A meta-analysis of individual patient data from randomized trials. Lancet.

[20] Carrero J.J., Trevisan M., Sood M.M., Bárány P., Xu H., Evans M., Friberg L., Szummer K. (2018). Incident atrial fibrillation and the risk of stroke in adults with chronic kidney disease: The Stockholm CREAtinine Measurements (SCREAM) Project. Clin. J. Am. Soc. Nephrol..

[21] Newsome B.B., Warnock D.G., Kiefe C.I., Weissman N.W., Houston T.K., Centor R.M., Person S.D., McClellan W.M., Allison J.J. (2005). Delay in time to receipt of thrombolytic medication among Medicare patients with kidney disease. Am. J. Kidney Dis..

[22] Powers W.J., Rabinstein A.A., Ackerson T., Adeoye O.M., Bambakidis N.C., Becker K., Biller J., Brown M., Demaerschalk B.M., Hoh B. (2018). 2018 guidelines for the early management of patients with acute ischemic stroke: A guideline for healthcare professionals from the American Heart Association/American Stroke Association. Stroke.

[23] Kelly D.M., Ademi Z., Doehner W., Lip G.Y., Mark P., Toyoda K., Wong C.X., Sarnak M., Cheung M., Herzog C.A. (2021). Chronic Kidney Disease and Cerebrovascular Disease: Consensus and Guidance From a KDIGO Controversies Conference. Stroke.

[24] Hankey G.J. (2003). Heart Outcomes Prevention Evaluation; Perindopril Protection Against Recurrent Stroke Study; Losartan Intervention for Endpoint Reduction in Hypertension Study. Angiotensin-converting enzyme inhibitors for stroke prevention: Is there HOPE for PROGRESS after LIFE?. Stroke.

[25] Ma C., Cao J., Lu X.C., Guo X.H., Gao Y., Liu X.F., Fan L. (2012). Cardiovascular and Cerebrovascular Outcomes in Elderly Hypertensive Patients Treated With Either ARB or ACEI. J. Geriatr. Cardiol..

[26] Shimizu Y., Maeda K., Imano H., Ohira T., Kitamura A., Kiyama M., Okada T., Ishikawa Y., Shimamoto T., Yamagishi K. (2011). Chronic Kidney Disease and Drinking Status in Relation to Risks of Stroke and its Subtypes: The Circulatory Risk in Communities Study (CIRCS). Stroke.

[27] Masson P., Webster A.C., Hong M., Turner R., Lindley R.I., Craig J.C. (2015). Chronic Kidney Disease and the Risk of Stroke: A Systematic Review and Meta-analysis. Nephrol. Dial. Transpl..

[28] Kelly D.M., Rothwell P.M. (2019). Does Chronic Kidney Disease Predict Stroke Risk Independent of Blood Pressure?: A Systematic Review and Meta-Regression. Stroke.

[29] Baigent C., Landray M.J., Reith C., Emberson J., Wheeler D.C., Tomson C., Wanner C., Krane V., Cass A., Craig J. (2011). The Effects of Lowering LDL Cholesterol with Simvastatin Plus Ezetimibe in Patients with Chronic Kidney Disease (Study of Heart and Renal Protection): A randomized placebo-controlled trial. Lancet.

[30] Tollitt J., Odudu A., Flanagan E., Chinnadurai R., Smith C., Kalra P. (2019). Impact of Prior Stroke on Major Clinical Outcome in Chronic Kidney Disease: The Salfort Kidney Cohort Study. BMC Nephrol..

[31] Kaze A.D., Jaar B.G., Fonarow G.C., Echouffo-Tcheugui J.B. (2022). Diabetic kidney disease and risk of incident stroke among adults with type 2 diabetes. BMC Med..

